# Prevalence of LA-MRSA in pigsties: analysis of factors influencing the (De)colonization process

**DOI:** 10.1038/s41598-022-21903-z

**Published:** 2022-10-26

**Authors:** Iris Kobusch, Iris Schröter, Sabrina Linnemann, Hannah Schollenbruch, Franka Hofmann, Marc Boelhauve

**Affiliations:** Department of Agriculture, South Westphalia University of Applied Sciences, 59494 Soest, Germany

**Keywords:** Antimicrobial resistance, Antibiotics

## Abstract

Professional pig husbandry is often associated with a more or less high load of LA-MRSA. Possible risk factors for LA-MRSA colonization in pig herds have already been identified in studies suggesting that housing conditions may affect LA-MRSA prevalence. In Europe, pigs are kept under variety of conditions. The aim of this study is to identify husbandry and housing condition factors that affect colonization with LA-MRSA. 78 pig farms were selected and assigned to three categories according to housing conditions: conventional, alternative and organic. Animal and surface samples were taken and examined for the presence of LA-MRSA at beginning and end of one fattening period per farm. Altogether, a significant (p < 0.05) decrease in colonization with LA-MRSA from beginning to end of the fattening periods in pigs and surfaces can be observed. Alternative farms showed a higher dynamic in the colonization. In organic farms, almost no colonization was found. Influencing housing condition factors that determine LA-MRSA status at the end of the fattening period are the number of pigs in the building, LA-MRSA status at the beginning of fattening period, material of the floor (straw bedding), strictness of black–white separation and antibiotic treatment during the fattening period. For pig farming in general, knowledge and measures to reduce the colonization with LA-MRSA would be important.

## Introduction

The use of antibiotics to treat bacterial infections in livestock husbandry is accompanied by the development of antibiotic resistance. Particular attention is paid to multidrug-resistant microorganisms (MDRO), especially livestock-associated methicillin-resistant *Staphylococcus aureus* (LA-MRSA) in pigsties. Thus, the evidence of MDRO in husbandries puts research in the focus of public interest, as a strict separation between human and veterinary medicine is not possible.

Studies identifying the co-occurrence of LA-MRSA positive pig herds and certain husbandry factors are already existent. Thus, potential risk factors for colonization have already been identified. There are carried out varying LA-MRSA-prevalences in pig herds depending on environmental parameters like herd size^1–3^ and type of production^1–3^ and herd type^4^. Other risk factors for LA-MRSA are antibiotic treatment^3,5,6^ and housing on slatted floor (in contrast to plain floor)^5^.

Reduction of antibiotic use in order to limit selective pressure is one strategy which has shown a positive effect in German and European pig production^7,8^. However, the causes for the development and spread of LA-MRSA appear to be complex and only partially understood. Some research suggests that pigs can change nasal LA-MRSA status during their lifetime^1,2,9–11^. Supported by own studies^12^, these changes will be further analyzed by the following study.

Previous studies have focused on identifying risk factors associated to MRSA colonization in pigsties^1–6^. The aim of this study is to identify housing condition factors that may promote decolonization of pigs and stable’s surfaces. A change in MRSA status from positive to negative is of particular interest in this context. Subject of the analyses are fattening pigs of one fattening period per farm. During this period the pigs in most farms stay in fixed groups with constant environmental (housing condition) factors. Nasal colonization of fattening pigs are animal individual tracked and selected surfaces in the stable (marked locations) were investigated at the beginning and the end of a fattening period. Before acquiring farms for sampling, a general subdivision is made into three housing systems: “conventional” (slatted floor, forced ventilation, closed building), “alternative” (straw bedding and/or outdoor climate, free ventilation, conventional slaughter pigs) and “organic” (according to guidelines of special organic associations, straw bedding and/or outdoor climate, free ventilation) husbandry. These housing systems summarize certain housing conditions. The intention is to collect samples on many different farms and focus less on the farm-specific level. Additional husbandry factors are recorded by data sheet during sampling visits to document each farm as completely as possible.

## Results

### LA-MRSA prevalence of pigs and surfaces

In total from beginning of the fattening period (visit I), 390 samples from fattening pigs and 430 samples from surfaces were taken in 78 farms. From the end of the fattening period (visit II), 366 animal samples and 427 surface samples were included. Overall, for both animal and surface samples, the load of LA-MRSA decreases from the beginning of fattening (visit I) to the end of fattening (visit II). In the animal samples, there was a reduction from 69.5 to 54.4%, and in the surface samples from 78.1 to 64.0%. The clonal complex CC398 is found in all samples that tested positive for MRSA.

### LA-MRSA prevalence of pigs and surfaces—conventional, alternative and organic farms

In the following evaluations, results of animals and surfaces are used that are available in both sampling dates (visit I and visit II). Thus, for each individual sample there is a value at the beginning (visit I) and at the end of the fattening period (visit II). Farms of the same housing type are considered as one group. All samples of pigs and surfaces are assigned to one of the three housing systems (conventional, alternative, organic). The pigs and the surfaces are exposed to similar housing conditions during the fattening period. The Mc Nemar test is used to determine whether the housing system and the environmental conditions during the fattening period have resulted in a change in the MRSA status of the animal or surface. Changes from positive to negative are of particular interest.

**Figure 1:**
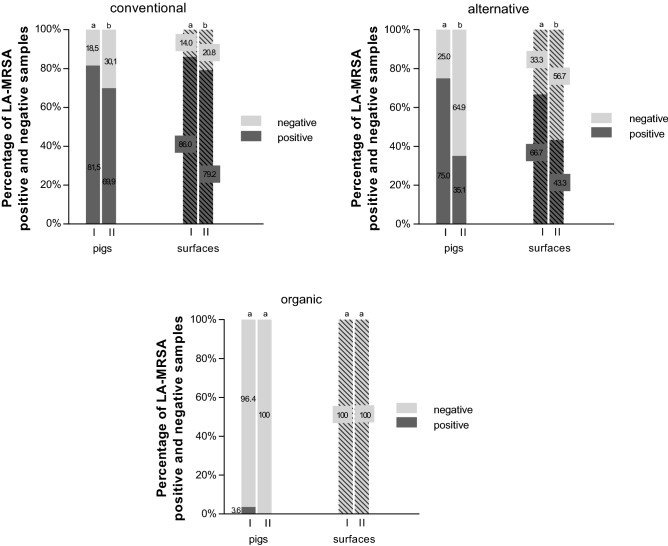
Percentage of LA-MRSA positive and negative samples at the beginning (I) and end of fattening period (II) in conventional (n = 2*256 pigs, n = 2*312 surfaces), alternative (n = 2*57 pigs, n = 2*60 surfaces) and organic (n = 2*53 pigs, n = 2*55 surfaces) housing systems; McNemar test, significant differences between visit I and II within the housing systems are indicated by letters (a,b) (p < 0.001).

55 conventional farms (71.0% of the analyzed farms in the study), 12 (15.0%) alternative farms and 11 (14.0%) organic farms are represented. Pigs of organic farms show very low colonization rates at the beginning of the fattening period (3.6%) in contrast to alternative (75.0%) and conventional farms (81.5%). In total, only two animals tested positive in organic farms for LA-MRSA at the beginning and no LA-MRSA is detectable in the samples from animals at the end of fattening period (Fig. 1). A reduction of LA-MRSA colonization can be observed in the samples from animals from conventional (81.5–69.9%) and alternative farms (75.0–35.1%) but with higher reduction in alternative farms. The detection of LA-MRSA shows a decrease in the animals and surface samples from the beginning to the end of the fattening period. In conventional and alternative housing systems, the proportion of positively tested surfaces at the end of the fattening period is higher than the proportion of positively tested pigs (Fig. 1). LA-MRSA is not detected on the surfaces in organic farms at any time. There is a decrease in LA-MRSA positive samples from surfaces in conventional and alternative housing systems. A reduction from the beginning to the end of the fattening period is also observed at the surfaces of the conventional (86.0–79.2%) and alternative (66.7–43.3%) housing systems (Fig. 1).

### LA-MRSA status of pigs and surfaces at the beginning and at the end of a fattening period

As for each animal and surface there is a MRSA status at the beginning of fattening and at the end of fattening, the change in status can be derived and classified into four categories (Table 1). The categories show the status (LA-MRSA-positive, LA-MRSA-negative) of the beginning of fattening period (visit I) and the end of fattening period (visit II). In this context, the pos-neg category is of particular interest, since decolonization of the animal or the surface has presumably occurred. For this reason, organic husbandry is not of interest in the further analyses on factors that might influence MRSA-(de)colonization processes, as only two MRSA-positive samples at the beginning of the fattening period and no positive samples at the end of the fattening period are found in total. Further conclusions on decolonization cannot be generated here due to this small amount of MRSA-positive samples.

**Table 1:** Categories of LA-MRSA status of the samples.

Category	Description
neg-neg (negative–negative)	Negative at beginning (visit I) and end (visit II) of fattening period
pos-neg (positive–negative)	Positive at beginning of fattening period (visit I) and negative at end of fattening period (visit II)
neg-pos (negative–positive)	Negative at beginning of fattening period (visit I) and positive at end of fattening period (visit II)
pos-pos (positive–positive)	Positive at beginning (visit I) and end (visit II) of fattening period

**Figure 2:**
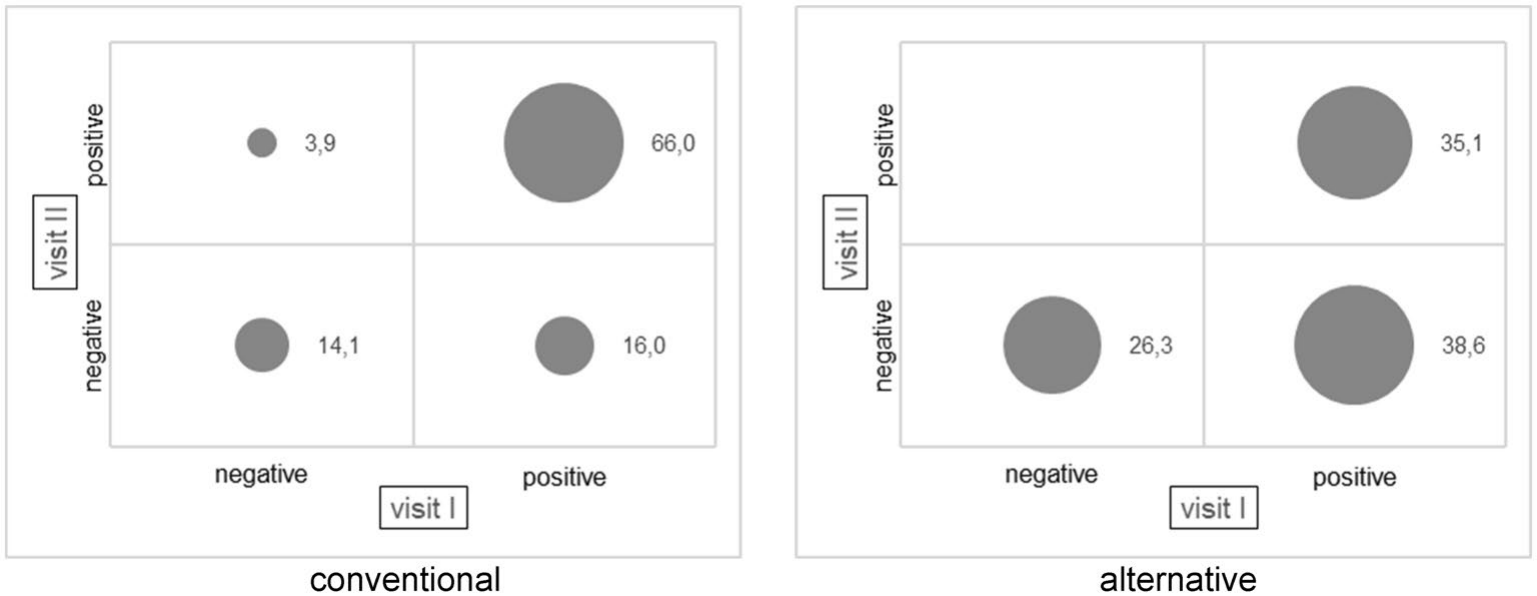
Categories of LA-MRSA-status of animals from beginning of fattening period (visit I) to end of fattening period (visit II) (percentages)—conventional (n = 2*256 = 512) and alternative (n = 2*57 = 114) housing systems.

Fattening pigs from conventional farms mainly have a positive nasal colonization with LA-MRSA at the beginning and end of fattening (66.0% pos-pos) (Fig. 2). In the alternative farms, the majority of fattening pigs are tested LA-MRSA-positive at the beginning of fattening and LA-MRSA-negative at the end of fattening (38.6% pos-neg). No animals show a shift from negative to positive in the alternative farms. However, there is a proportion of 35.1% pos-pos.

**Figure 3:**
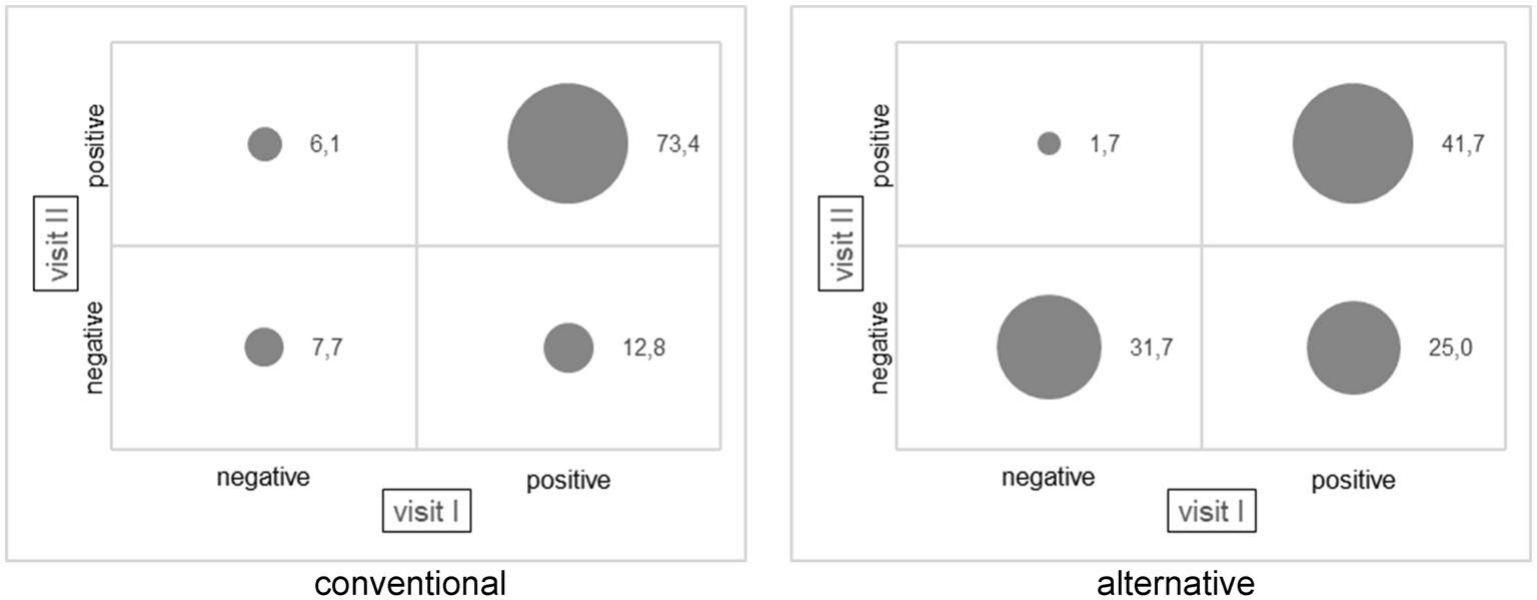
Categories of LA-MRSA-status of surfaces from beginning of fattening period (visit I) to end of fattening period (visit II) (percentages)—conventional (n = 2*312 = 624) and alternative (n = 2*60 = 120) housing systems.

66.1% of the samples from surfaces from conventional housing systems are categorized as pos-pos (Fig. 3). In the alternative housing systems, the majority of the samples from surfaces, 41.7%, are found in the pos-pos category. For conventional and alternative husbandries, the observed frequency differs significantly (Chi^2^ test, p < 0.05) from the expected frequency in animals and surfaces.

Important indications for approaches to decolonization of pig herds are given by the results from the pos-neg category. 16.0% of the samples from pigs of conventional housing systems are LA-MRSA-positive at the beginning of the fattening period and LA-MRSA-negative at the end (Fig. 2). 12.8% of the associated surfaces show the corresponding status (Fig. 3). 38.6% of the animals’ samples and 25.0% of surfaces’ samples from alternative husbandries are pos-neg.

### Consideration of individual housing condition factors

In both conventional and alternative husbandry, decolonization occurs in part of the animals. Differences between the housing systems are detectable in the results. In addition to the recording of the housing system, the sampling was accompanied by a recording of specified housing conditions by the samplers (content of the recording see Data availability and Competing interests). For a more detailed consideration of specific housing condition factors, a logistic regression analysis is carried out. The effect of the existing housing conditions on the MRSA status at visit II is considered, since the pigs are exposed to these conditions during the fattening period. It is investigated whether certain housing conditions factors can affect the MRSA status at the end of the fattening period (visit II). Thus, the values in Fig. 2 are to be taken in relation to the LA-MRSA status of pigs at the end of the fattening period (visit II) as dependent variable. In the following analyses are farms of the conventional and the alternative type included. Organic farms were not considered further, since a development from positive to negative occurred only in a total of two pigs. The logistic regression model was significant with Chi^2^ (5) = 135.02, p < 0.001 (n = 312). McFadden’s Pseudo R^2^ amounted to 0.33 indicating an excellent model fit^13^.

In the present study, five factors were identified that could influence LA-MRSA status (Table 2). The more pigs were kept in the building, the higher the probability that the animals tested positive for LA-MRSA at the end of the fattening period (visit II). If pigs tested positive for LA-MRSA at the beginning of the fattening period (visit I), the probability of testing positive at the end of the fattening period (visit II) is higher than if the animals tested LA-MRSA negative at the beginning of the fattening period (visit I). If pigs are kept on straw bedding, they are less likely to show nasal colonization at the end of the fattening period (visit II). This statement is equally valid for a strict black-white separation (strictness of separation of the paths of pig husbandry and non-pig husbandry as a biosafety barrier).

**Table 2:** Results of the logistic regression model analyzing the influence of predictor variables on LA-MRSA status at visit II (end of fattening period).

Predictor variables		Coefficient	[95% Conf. interval]		*p*-value
Number of animals	Continuous (per 100)	0.16	0.07	0.25	< 0.001
LA-MRSA status visit I	Negative	base			
	Positive	2.93	2.09	3.77	< 0.001
Floor	No straw bedding	base			
	Straw bedding	− 2.17	− 3.08	− 1.25	< 0.001
Black–white separation*	No clear separation	base			
	Clear separation	− 1.41	− 2.23	− 0.59	0.001
Antibiotic treatment	No antibiotic treatment	base			
	Antibiotic treatment	1.16	0.39	1.93	0.003

*= separation of the paths of pig husbandry and non-pig husbandry.

**Figure 4:**
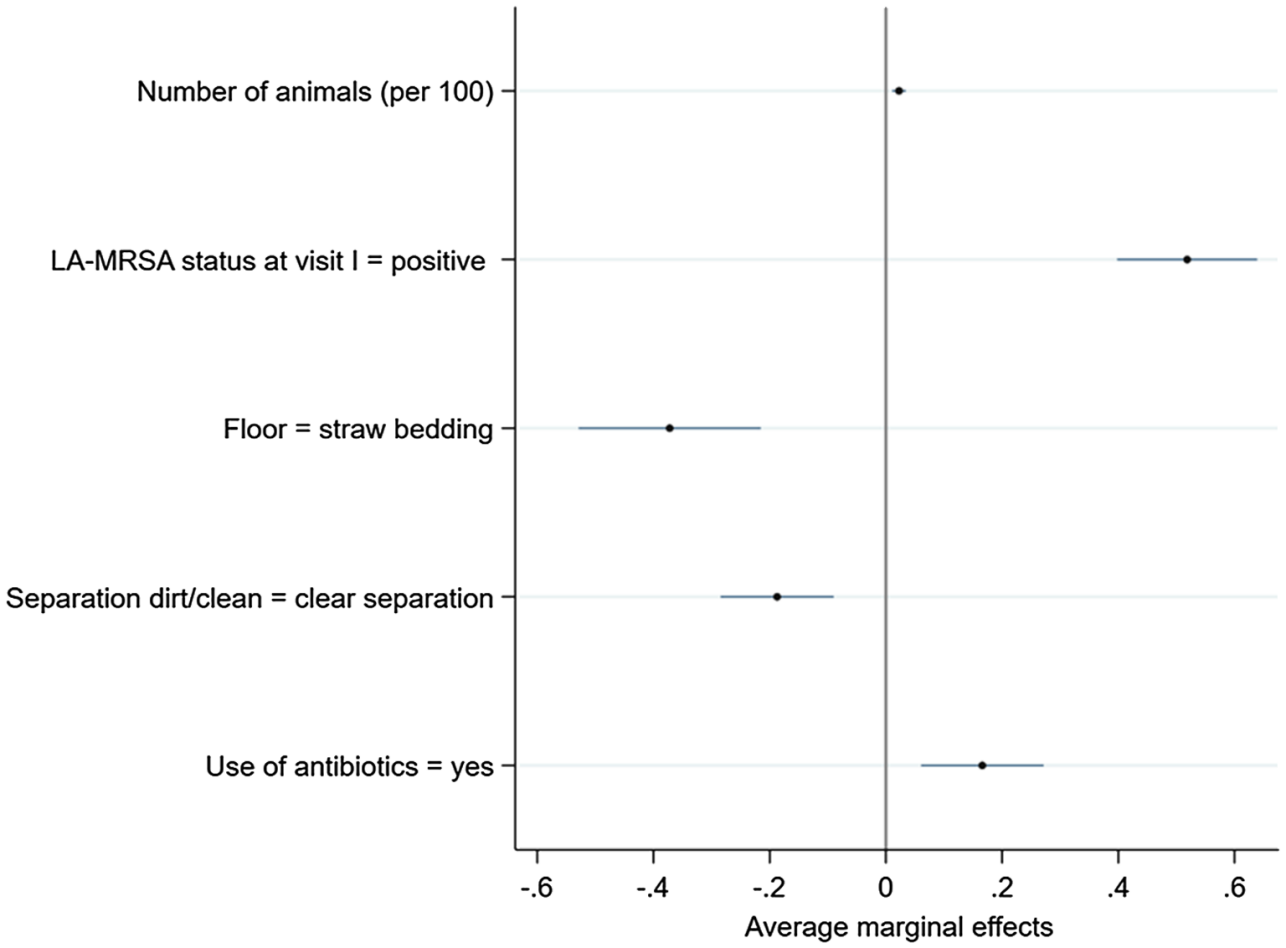
Estimated average marginal effects (points) with 95% CI (lines) of the independent variables on the probability of being LA-MRSA positive at visit II.

To make the extent to which the predictor variables influenced the probability of being LA-MRSA positive at the end of the fattening period (visit II) more tangible, average marginal effects can be consulted (Fig. 4). The probability of being LA-MRSA positive at the end of the fattening period increased on average by 2.3% if the number of animals increased by one unit, i.e. by 100 animals. The probability of being LA-MRSA positive at the end of the fattening period increased by 51.9% if the status at visit I was LA-MRSA positive, decreased by 37.2% if the animals were kept on straw bedding, decreased by 18.7% if there was a clear separation between the dirt and the clean area and increased by 16.6% if the animals were treated with antibiotics during the fattening period.

## Discussion

One fattening period in each of 78 farms was sampled. A wide range of pig housing systems were examined. According to defined criteria, these farms were categorized in three housing systems (conventional, alternative, organic). More conventional (n = 58) than alternative (n = 12) and organic (n = 11) farms were investigated. The imbalance could not be equalized in these studies because alternative and organic pig farming is quite rare in Germany. 96% of the pigs are kept on partially slatted or fully slatted floor^14^. However, a larger number of alternative and organic farms would have been preferable in order to obtain a more precise information. Sampling was carried out on individual tracked pigs and surfaces in the barn at the beginning and at the end of the fattening period. The selected age section of the animals and the two time points offer the advantage of constant stable environmental conditions before and after sampling (constant conditions in the rearing stable and constant conditions in the fattening stable). The relocation from piglet rearing to the fattening stable represents a change in environmental conditions (e.g. change of the building, new group composition, underground, air ventilation systems, water and feeding system). In our own studies^12,15^ it was found that the colonization of the animals and the surfaces adapts to the changed conditions over a period of several weeks after being housed in a new stable (with new environmental conditions).

The aim of the study was to sample as large a number of farms as possible in order not to highlight the influence of the individual farm too strongly. For reasons of work economy, the number of samples per farm had to be reduced. Increasing the number of samples per farm would probably strengthen the conclusions of the study.

Colonization varies between farm types as well as between the two sampling times during the fattening period. In the conventional farms there was a reduction in the rate of LA-MRSA positive samples from the beginning (81.5% LA-MRSA positive samples of pigs, 86.0% LA-MRSA positive samples of surfaces) to the end (69.9% LA-MRSA positive samples, 79.2% LA-MRSA positive samples of surfaces). At the end of the fattening period, the analyzed surfaces show a higher rate of LA-MRSA positive samples than the animals. Similar results could already be found in own previous studies^12,15^. Here, it was found out that the contamination with LA-MRSA on surfaces and in the air in the barn depends on LA-MRSA status and development of the housed pig^15^. Conventional and alternative husbandries showed a high proportion of LA-MRSA positive samples, whereas almost no LA-MRSA was detectable in the organic farms. These results are consistent with those found in the literature^16–18^. One possible reason for the low colonization rates at the beginning of fattening on organic farms is that these farms have to use piglets from organic rearing systems as far as possible^19,20^. In the conventional and alternative farms, piglets from conventional farms were housed in.

As the focus of this study was on the change of colonization of LA-MRSA status during the fattening period, four categories were generated from the results of sampling at the beginning and end of fattening. From the viewpoint of the OneHealth concept, special attention is paid to the pos-neg category (LA-MRSA positive at the start of fattening and LA-MRSA negative at the end of fattening) of the pigs examined. The proportion of these switchers was highest in alternative housing systems. A change took place in 38.6% of the animals in these systems (compared to conventional husbandry (16.0%). Of particular interest are influencing factors that cause decolonization of animals (and decontamination of surfaces) during the fattening period in both systems (conventional and alternative). A first approach to identify factors influencing the LA-MRSA status of the examined animals was offered by statistical analysis. The recorded husbandry factors were taken and related to the LA-MRSA status at the end of the fattening period. The statistical analysis reveals five housing condition factors that have an influence on the LA-MRSA status at the end of fattening. In particular, the number of pigs per housing unit, the LA-MRSA status at the beginning of the fattening period and housing on straw bedding can be highlighted.

There are very few results in the literature on the development of LA-MRSA status over the course of fattening with simultaneous recording of housing condition factors. Thus, there is rarely any knowledge about the circumstances under which colonization with LA-MRSA occurs. Some authors^1–6^ have already mentioned risk factors for colonization. Measures to decolonize animals have also been mentioned (intensive C&D measures, feeding additives, spraying of vegetable and essential oils) but have not been successful so far^3^. In the above-mentioned study by Brockers^3^, however, increasing detection rates of LA-MRSA were recorded in the first 2 weeks after entry into a conventional fattening house. Subsequently, a stabilization to a stable level could be observed. Own results in stables with straw bedding have shown decreasing colonization in pigs during the fattening period^12^.

Blaha et al.^16^ and van de Vijver et al.^17^ have already found out that there is less colonization of animals with LA-MRSA in alternative and organic husbandries. Alt et al.^21^ note the incidence of LA-MRSA correlates positively not only with the density of conventional pig husbandries in a region, but also with farm size. Additionally, Cuny et al.^18^ detected that the incidence of LA-MRSA is lower on farms with alternative farming methods.

The results of this study indicate that straw bedding in combination with other factors (low number of animals per unit, negative LA-MRSA status at the beginning, black–white separation, no use of antibiotics) has a positive influence on the decolonization of fattening pigs. Possibly, there could be created a complex environmental microbiota with bacterial competition caused by straw bedding (alternative husbandry) and provides apparently a competitive flora. However, straw bedding might affect MRSA status in synergy with other environmental factors that could not be separated from the straw factor in the analysis. In this study, all farms with straw bedding also had access to free air. This means that either an outdoor paddock was available for the pigs or there was no forced ventilation of the barn.

Approaches for decolonizing pigs could already start in piglet production, since in agricultural practice a switch from closed stables with slatted floor to straw bedding cannot be realized in a short period of time. In addition, studies indicate that the colonization of the sow already has an influence on the LA-MRSA status of the piglets. Early contact with methicillin-sensitive *Staphylococcus aureus* (MSSA) could also play a role^10,11,22^. Whereby Fetsch et al.^22^ found that MSSA and MRSA could also occur simultaneously in the nose of pigs.

This study investigates the influence of housing conditions on changes in MRSA status during the fattening period of pigs to better understand which factors may influence MRSA status and to define effective strategies to reduce MDRO occurrence in livestock breeding. Here, the investigations involve the nasal colonization of LA-MRSA in individually tracked fattening pigs at the beginning and end of the fattening period, including the associated surfaces in the barn. The identification of housing factors that support a change of LA-MRSA status is interesting with regard to identify possibilities for decolonization. In particular, the change from LA-MRSA positive to negative is a point of interest. Opportunities for prevention and decolonization are a particular focus. This can create opportunities for reducing resistant pathogens in the food chain. In summary, it is not one particular husbandry system that is at an advantage in determining decolonization of MRSA. Rather, it is a package of husbandry factors, like straw bedding, use of antibiotics, number of animals in the stable and a black–white-separation as biosafety barrier that can promote decolonization.

## Methods

### Aims of the study

This study is being implemented in the context of epidemiological studies on the zoonotic spread of bacterial pathogens with antibiotic multiresistance. LA-MRSA is known to be less prevalent on organic farms. However, this does not provide immediate solutions for farms with already colonized pigs. An intervention to decrease the occurrence of MDRO by national authorities is the reduction of antibiotic doses. Further possibilities for limiting the spread of LA-MRSA are to be indicated by this study. Housing condition factors that may promote decolonization of pigs and stable’s surfaces during a certain period should be found. Particularly the effects of conventional and alternative housing conditions in pig fattening on the occurrence of LA-MRSA were investigated. Deeper analyses of potential influences e.g. straw bedding material, the execution of cleaning and disinfection, hygiene interventions and space per animal were in the focus of the investigations.

### Pig fattening farms

78 farms with pig fattening, 55 conventional, 12 alternative and 11 organic husbandries, were visit from March 2018 to September 2020. The characterization of “conventional”, “alternative” and “organic” farming systems based on certain housing condition factors. Conventional farming is defined by a husbandry with slatted or partially slatted floor in the pen, forced ventilation and closed buildings. Alternative farming includes husbandry on straw bedding and/or outdoor climate in the stable and/or free ventilation but with a classification to conventional husbandry and conventional slaughter pigs. Organic farming works according to the guidelines of special associations (“Bioland”, “Naturland” etc.) or other organizations (e.g. guidelines of the EU) and includes straw bedding and/or outdoor climate in the stable and free ventilation. Participating farms were selected by approaching existing contacts of the project partners and networks.

### Sampling environment and animals

A fattening period of one herd was chosen per farm. Sampling took place at two occasions in one pen of a compartment. The first sampling was within 1–14 days after housing in (beginning of the fattening period, visit I), second sampling followed at the end of the fattening period (10–14 weeks after housing in, visit II). These two dates are chosen because the animals are exposed to constant housing conditions for at least six weeks during the rearing period before fattening, and during the fattening period of 10–14 weeks, constant conditions are also given. Housing in to the fattening stable represents a move to a different stable with new housing conditions and a new animal group composition.

Ethical review and approval was waived for this study, because according to the guidelines in force at that time of the Department of Agriculture, South Westphalia University of Applied Sciences, this type of research does not require approval. Animals are not harmed or adversely affected by participating in the study. All experiments and methods were performed in accordance with relevant guidelines and regulations. Sampling was carried out as part of standard farm practices for the grooming of animals. The owners of the animals were informed in writing of the procedure prior to the studies and voluntarily agreed to participate in the studies. Permission was provided by the farm owners for the use of animal samples and sampling was performed in the presence of the animal owners.

Samples were taken from the pen’s/stable’s surfaces by using a protocol with defined and marked sampling locations (exact sampling locations can be taken from Table 3) and from five individually marked and tracked animals in the pen. Surface LA-MRSA samples were taken by using sponges measuring 5 cm × 10 cm (Polywipe™, Check Diagnostics GmbH, Westerau, Germany). These were already moistened in their containers by a phosphate buffer. The sponge was swabbed approximately 30 cm of the surface, resulting in a sampled area of approximately 300 cm^2^.

**Table 3:** Number of samples and sampling locations per farm visit.

	No. of samples	Location	Type of sample
Surfaces	2	Vertical surface in animals’ range (wall of the pen)vertical surface in animals’ range (wall of the pen)	Sponge swab
	2	Horizontal surface outside animals’ range	
	1	Exhaust air duct*	
	1	Optional sample (vertical or horizontal surface)	
Animals	5	Nasal atria	Nasal swab

*If present.

Nasal LA-MRSA carriage of the pigs was analyzed by using swabs with Amies transport medium (VWR, Langenfeld, Germany). The swab was taken in rotating movements without touching the outside of the snout. Pigs were individually marked for identification for the second sampling.

### Bacterial culturing

Tryptic Soy Broth TSB (Merck, Darmstadt, Germany) + 6.5% NaCl (VWR, Langenfeld, Germany) was used to enrich LA-MRSA from animal nasal swabs and dust samples. The nasal swabs were transferred to culture tubes containing 9 ml of this culture medium and were incubated at 37 °C for 18 h. Next, 500 µl of sample was transferred into 5 ml of TSB+Cefoxitin/Aztreonam C/AZ (Mediaproducts, Groningen, Netherlands) and incubated at 37 °C for 18 ± 2 h. This step was followed by inoculating 10 µl onto chromID^®^ MRSA SMART agar (Biomérieux, Nurtingen, Germany) and incubation at 37 °C for 24 h. As in the following descriptions, the agar plates were evaluated according to the manufacturer’s instructions.

The sponges of the dust samples were filled up with 50 ml TSB + 6.5% NaCl in sterile blender bags, blendered for 30 sec at 230 rpm, and then incubated at 37 °C for 18 h. 500 µl of the sample was transferred to 5 ml TSB+C/AZ (Mediaproducts, Groningen, Netherlands). Incubation at 37 °C for 18 ± 2 h was subsequently performed. The following day, 10 µl was spread on chromID^®^ MRSA SMART (Biomérieux, Nurtingen, Germany) and incubation at 37 °C for 24 h was done. Evaluation of the plates was based on the manufacturers’ instructions.

Typing of MRSA has been performed before following a previously described protocol^23^.

### Datasheet for recording housing conditions

Datasheets were recorded for each of the two farm visits and sampling. The scheme of the data collection of the first visit (visit I) contained:

- Specifications of the farm: size and structure, genetic of the pigs;
- Husbandry system: size and structure, air volume, ventilation and feeding technique;
- Feed and water: feed components, zinc and acid content, feed additives, water chlorination;
- Organic material and bedding material;
- Animal health and hygiene: hygiene sluice, black-white-separation (biosafety barrier, separation of the paths of pig husbandry and non-pig husbandry), contact to other animals, handling of sick animals, use of antibiotics, cleaning & disinfection: procedure, active substances.

The datasheet of the first visit was intended to record the general management of the fattening pigs, while the datasheet at the end of fattening period (visit II) was intended to identify any deviations from this. The second datasheet contained similar or identical questions in a shortened form, such as how the animals were actually fed, which organic materials were used and how often they were replaced, and whether and which medications had to be used (Table S1).

### Statistical analyses

For analyses of the nominal data and the non-parametric variables, the McNemar test and the Chi^2^ test were used to test for significant differences between samples from beginning (visit I) and end of fattening period (visit II) (McNemar test) and between categories (Chi^2^ test). For statistical analyses, the software SPSS Statistics 27 (IBM Deutschland GmbH, Ehningen, Germany) were used.

Furthermore, statistical analyses were conducted with Stata 16.1 (StataCorp LLC 2019). To answer the question whether the assumed predictor variables number of animals (continuous, per 100 animals), LA-MRSA status at visit I (positive/negative), floor characteristics (straw/no straw), separation between dirt and clean area (clear separation/no clear separation) and antibiotic treatment (treatment/no treatment) influenced the LA-MRSA status at visit II (positive/negative) a logistic regression model was used. In these analyses, only pigs kept in conventional production systems were considered. Organic farms were excluded since organic production systems usually differ greatly from conventional production systems due to legal requirements.

## Supplementary Information

Supplementary Table S1.

## Data Availability

The data presented in this study are available on request from the corresponding author.
