# Supplementary material for: Prevalence of LA-MRSA in pigsties: analysis of factors influencing the (De)colonization process
Source: Sci Rep. 2022 Oct 26;12:18000. doi: 10.1038/s41598-022-21903-z (PMC9605981; doi:10.1038/s41598-022-21903-z)
Supplement: Supplementary file 1 — Supplementary Table S1. [file 41598_2022_21903_MOESM1_ESM.pdf]

# Prevalence of LA-MRSA in Pigsties - Analysis of Factors influencing the (De)colonization Process

**Iris Kobusch [1,*], Iris Schröter [1], Sabrina Linnemann [1], Hannah Schollenbruch [1], Franka Hofmann [1] and Marc Boelhauve [1]**

[1] Department of Agriculture, South Westphalia University of Applied Sciences, 59494 Soest, Germany; kobusch.iris@fh-swf.de (I.K.); schroeter.iris@fh-swf.de (I. S.); linnemann.sabrina (S.L); schollenbruch.hannah@fh-swf.de (H.S.); hofmann.franka@fh-swf.de (F. H); boelhauve.marc@fh-swf.de

[*] Corresponding author: kobusch.iris@fh-swf.de

| Category | Recorded data | Time of data collection |
| --- | --- | --- |
| Specifications of the farm | 1.1 Production system/farm organization<br>1.2 Total number of fattening places<br>1.3 Number of fattening places on site<br>1.4 Number of animals in the building<br>2.1 Conventional - organic<br>2.2 Region/county<br>3.1 Participation in marketing programs<br>4.1 QS participation<br>5.1 No. of caretakers<br>6.1 Other animals in the building<br>8.1 Conv./organic piglets<br>8.2 Genetics<br>9.1 House weight<br>9.2 Days since housing / 71.2 Days since housing II<br>71.1 Animal weight II | visit I<br>visit I<br>visit I<br>visit I<br>visit I<br>visit I<br>visit I<br>visit I<br>visit I<br>visit I<br>visit I<br>visit I<br>visit I<br>visit I + II<br>visit II |
| Husbandry system | 10.1 Re-housing during the fattening period / 72.1 Re-housing during fattening period II<br>10.2 Reason for re-housing / 72.2 Reason for re-housing II<br>10.3 Time of re-housing / 72.3 Time of re-housing II<br>29.1 Free or forced ventilation<br>30.1 Forced ventilation, supply air<br>31.1 Forced ventilation, exhaust air<br>32.1 Free ventilation, airflow<br>33.1 Outdoor pen<br>33.2 Intensity of usage outdoor pen<br>50.1 Room volume bay<br>51.1 Room volume compartment<br>52.1 Stocking density bay<br>53.1 Stocking density compartment<br>57.1 Separate-sex boar fattening<br>59.1 Temperature inside compartment<br>59.2 Temperature outside<br>60.1 Humidity in compartment<br>60.2 Humidity outside<br>61.1 Floor material<br>62.1 Material bay and compartment partitions<br>63.1 Material feeding troughs<br>64.1 Outdoor pen floor material<br>65.1 Outdoor pen walls material<br>66.1 Outdoor pen floor material<br>67.1 Percentage of closed floor area in the pen<br>68.1 Condition of outer stable<br>69.1 Condition of housing equipment | visit I + II<br><br>visit I + II<br>visit I + II<br>visit I<br>visit I<br>visit I<br>visit I<br>visit I<br>visit I<br>visit I<br>visit I<br>visit I<br>visit I<br>visit I<br>visit I<br>visit I<br>visit I<br>visit I<br>visit I<br>visit I<br>visit I<br>visit I<br>visit I<br>visit I<br>visit I<br>visit I<br>visit I |
| Feed and water | 13.1 Feed components (growing phase)<br>14.1 Ready-mix feed (growing phase)<br>14.2 Grain supplement (growing phase)<br>15.1 Ready-mix feed (finishing phase) / 74.1 Ready-mix feed (finishing phase) II<br>15.2 Grain supplements (finishing phase) / 74.2 Grain supplement (finishing phase) II<br>16.1 Dry or liquid feeding<br>17.1 Zinc content growing pig feed<br>17.2 Zinc content finishing pig feed / 75.1 Zinc content finishing pig feed II<br>18.1 Duration of feed storage / 76.1 Duration of feed storage II<br>19.1 Use of acids / 77.1 Use of acids II<br>19.2 Product name acid / 77.2 Product name acid II<br>19.3 Acid concentration in product / 77.3 Acid concentration in product II<br>20.1 Use of additional components / 78.1 Use of additional components II<br>20.2 Additional components / 78.2 Additional components II | visit I<br>visit I<br>visit I<br>visit I + II<br><br>visit I + II<br><br>visit I<br>visit I<br>visit I + II<br><br>visit I + II<br>visit I + II<br>visit I + II<br>visit I + II<br>visit I + II<br>visit I + II |

| | | |
|---|---|---|
| | 20.3 Used quantity additional components / 78.3 Used quantity additional components II<br>21.1 Chlorine use / 79.1 Chlorine use II<br>21.2 Used concentration chlorine / 79.2 Used concentration chlorine II<br>79.3 Product name chlorine dioxide II<br>73.1 Feed components (finishing phase) II | visit I + II<br><br>visit I + II<br>visit I + II<br>visit II<br>visit II |
| Organic employment material and bedding material | 22.1 Presence of bedding material<br>23.1 Bedding material<br>24.1 Duration of storage bedding material / 80.1 Duration of storage bedding material II<br>25.1 Refill interval of bedding material in barn / 81.1 Refill interval of bedding material stable II<br>25.2 Refill interval of bedding material in outdoor pen / 81.2 Refill interval of bedding material outdoor pen II<br>82.1 Date of last refill of bedding material II<br>58.1 Bedding height<br>26.1 Organic employment material / 83.1 Organic employment material II<br>27.1 Frequency of exchange organic employment / 84.1 Frequency of exchange organic material II<br>28.1 Storage of organic employment and bedding material<br>85.1 Date of last refill/exchange organic employment II<br>86.1 Storage org. employment material and bedding material II | visit I<br>visit I<br>visit I + II<br><br>visit I + II<br><br>visit I + II<br><br>visit II<br>visit I<br>visit I + II<br>visit I + II<br><br>visit I<br>visit II<br>visit II |
| Animal health and hygiene | 5.2 Number of employees with contact to other farms<br>7.1 Piglet transporter<br>11.1 Strictness of black-white separation (hygiene system)<br>12.1 Contact to other animals<br>12.2 Contact to other listed animal species<br>34.1 Cleaning procedures (C&D)<br>35.1 soaking (C&D)<br>35.2 duration of soaking (C&D)<br>36.1 draining slurry (C&D)<br>37.1 wet-cleaned parts of the bay<br>38.1 disinfectant<br>39.1 duration of vacancy (bay)<br>40.1 all in-all out procedure<br>41.1 feed storage cleaning / 87.1 feed store cleaning II<br>41.2 reason for cleaning feed storage / 87.3 reason for cleaning feed store II<br>87.2 Date of cleaning feed store II<br>42.1 Administration of antibiotics / 88.1 use of antibiotics II<br>43.1 Use of antibiotics in the past 14 days<br>43.2 Antibiotics administered<br>89.1 Use of antibiotics2 II<br>89.2 Product name of antibiotic II<br>90.1 Withdrawal period medication II<br>90.2 Product name medication (withdrawal period) II<br>44.1 Withdrawal period for medication<br>44.2 Used medications<br>45.1 Handling of sick animals<br>46.1 ADG (average daily gain) last 3 fattening periods<br>47.1 Loss rate last 3 fattening periods<br>48.1 IXP/kg SW last 3 fattening periods<br>49.1 Abnormal findings at slaughterhouse<br>54.1 Proportion of animals with puny growth<br>55.1 Proportion of heavily fecally soiled animals<br>56.1 Proportion of animals with lesions of skin/ear/tail<br>70.1 Clear black-white separation II (hygiene system)<br>91.1 Whereabouts of sick animals II<br>92.1 Particular health problems II | visit I<br>visit I<br>visit I<br>visit I<br>visit I<br>visit I<br>visit I<br>visit I<br>visit I<br>visit I<br>visit I<br>visit I<br>visit I<br>visit I + II<br>visit I + II<br><br>visit II<br>visit I + II<br>visit I<br>visit I<br>visit II<br>visit II<br>visit II<br>visit II<br>visit I<br>visit I<br>visit I<br>visit I<br>visit I<br>visit I<br>visit I<br>visit I<br>visit I<br>visit I<br>visit II<br>visit II<br>visit II |
